# Longitudinal Microbiome Analysis in a Dextran Sulfate Sodium-Induced Colitis Mouse Model

**DOI:** 10.3390/microorganisms9020370

**Published:** 2021-02-12

**Authors:** Hyunjoon Park, Soyoung Yeo, Seokwon Kang, Chul Sung Huh

**Affiliations:** 1 Research Institute of Eco-Friendly Livestock Science, Institute of Green-Bio Science and Technology, Seoul National University, Pyeongchang, Gangwon 25354, Korea; hyunjoons@snu.ac.kr; 2 Advanced Green Energy and Environment Institute, Handong Global University, Pohang 37554, Korea; 3 Department of Agricultural Biotechnology, College of Agriculture and Life Sciences, Seoul National University, Seoul 08826, Korea; ysso1215@gmail.com; 4 Department of Life Sciences, Handong Global University, Pohang 37554, Korea; 21837003@handong.edu; 5 Graduate School of International Agricultural Technology, Seoul National University, Pyeongchang, Gangwon 25354, Korea

**Keywords:** intestinal microbiota, DSS-induced colitis development, longitudinal studies, dynamics, susceptibility, gut microbes

## Abstract

The role of the gut microbiota in the pathogenesis of inflammatory bowel disease (IBD) has been in focus for decades. Although metagenomic observations in patients/animal colitis models have been attempted, the microbiome results were still indefinite and broad taxonomic presumptions were made due to the cross-sectional studies. Herein, we conducted a longitudinal microbiome analysis in a dextran sulfate sodium (DSS)-induced colitis mouse model with a two-factor design based on serial DSS dose (0, 1, 2, and 3%) and duration for 12 days, and four mice from each group were sacrificed at two-day intervals. During the colitis development, a transition of the cecal microbial diversity from the normal state to dysbiosis and dynamic changes of the populations were observed. We identified genera that significantly induced or depleted depending on DSS exposure, and confirmed the correlations of the individual taxa to the colitis severity indicated by inflammatory biomarkers (intestinal bleeding and neutrophil-derived indicators). Of note, each taxonomic population showed its own susceptibility to the changing colitis status. Our findings suggest that an understanding of the individual susceptibility to colitis conditions may contribute to identifying the role of the gut microbes in the pathogenesis of IBD.

## 1. Introduction

Inflammatory bowel disease (IBD) is a comprehensive disorder that encompasses various types of chronic inflammations in the gastrointestinal (GI) tract, depending on the occurrence location and inflammatory severity. IBD has a multifactorial pathogenesis that results from the complex interplay of genetic predispositions, host immune response, environmental factors, and gut flora [1,2]. In recent decades, gut dysbiosis, which refers to the structural and/or functional imbalance of the GI tract microbiome, has been elucidated in IBD patients, and it has been postulated that intestinal microflora play a key role in the pathogenesis of IBD [3,4]. It has been known that stimulation of the immune system and gut dysbiosis are primarily associated with the development of chronic inflammation in the late stages of IBD [5]. Emerging studies in germ-free mice have demonstrated that gut microbes contribute to colitis conditions [6,7,8]. Therefore, microbiome-based therapeutics that modulate the gut ecology have been proposed as a novel strategy for preventing or alleviating IBD [5,9].

The gut microbiota composition changes are dynamic and relatively immediate, influenced by various factors such as diet, medication, stress, and hygiene [10,11]. The dynamics of fluctuating gut microbiota are even more severe in IBD patients due to recurrent active disease and remission states [12]. Numerous microbiome studies have been primarily conducted as a cross-sectional study to identify differences in microbial compositions between healthy controls and IBD patients [13,14,15]. However, the microbiome results are indecisive and inconsistent among individual studies because the cross-sectional outcomes do not reflect the dynamics of gut microbiome that depend on highly personalized disease status [12,16,17,18,19]. Therefore, longitudinal microbiome observations from onset to development of the disease are required to reveal the gut microbes which cause or contribute to IBD pathogenesis, but there is a limit to conducting such an approach on humans.

Experimental models of IBD are useful tools to improve the understanding of the mechanistic relations of intestinal microorganisms with IBD pathogenesis. The most representative is the dextran sulfate sodium (DSS)-induced colitis model because it is simple to induce colonic inflammation and similar to ulcerative colitis in pathology, etiology, and therapeutic response [20,21]. DSS is directly toxic to the intestinal epithelium, resulting in disruption of mucosal barrier function and stimulation of the immune response [22,23]. Studies in DSS-induced colitis mouse models in which intestinal bacteria are eliminated by using antibiotics have shown that gut microbes contribute to the onset of inflammation in acute cases rather than chronic cases [24]. According to Brinkman et al. [25], in a genomic modified DSS-resistant mouse model, the gut microbiota affected acute colitis susceptibility regardless of host genotype. Thus, the acute DSS-induced colitis model is suitable for studying intestinal mucosal barrier dysfunction, concomitant microbial dysbiosis, and their role in IBD pathogenesis [26,27,28]. However, as with human studies, there are still limitations. The gut microbiome results are varied and inconsistent among studies due to the different colitis conditions, including the dose and duration of DSS application. For instance, the expansion of *Proteobacteria* has been commonly reported in the DSS-induced colitis model [29,30], but its expansion was not observed under mild colitis status [28]. In addition, population reduction was detected in mice treated with 5% DSS for 14 days [31]. As an example, at the genus level, the increasing or decreasing abundances of *Lactobacillus*, *Prevotella*, and *Parabacteroides* also indicated a discrepancy between the results [29,30,32,33,34].

Therefore, in the present study, we observed the cecal microbiota changes in a mouse in vivo colitis model based on serial DSS dose and duration, identified colitis-associated bacterial taxa that can be used as potential microbial markers of gut dysbiosis in DSS-induced colitis, and investigated the susceptibility of the individual taxa to colitis indicators to provide an understanding of their role in IBD pathogenesis.

## 2. Materials and Methods

### 2.1. A Two-Factor Design for an Experimental Mouse in Vivo Colitis Model

One hundred and twelve six-week-old female C57BL/6J mice were purchased from Daehan Bio Link Co., Ltd., South Korea, which adopted the strain in 2010 from Taconic Farms, Inc. Mice were maintained in a controlled animal facility under specific pathogen-free conditions. This study was carried out in accordance with the guidelines established by the Korean Association for Laboratory Animals. The protocol was approved by the Committee on the Ethics of Animal Experiments of Handong Global University (approval No. 20190424-008). The in vivo experiment was carried out in a 4 × 7 design, which had two factors (DSS concentration and duration), each with four and seven levels, respectively. After stabilization for two weeks, mice were divided into four groups (*n* = 28 per group), and colitis was induced by administration of DSS (molecular weight 36 to 50 kDa; MP Biomedicals, Santa Ana, CA, USA) at 1%, 2% and 3% concentrations in drinking water ad libitum. The 0% DSS group was provided with only the vehicle, which was autoclaved distilled water (DW). Each treatment group was kept in the same cage with enough space to accommodate all of the mice to avoid ‘cage effects’ on the composition of the gut microbiota [35,36]. The experiment lasted 12 days, and four mice from each DSS dose group were randomly selected, weighed, and sacrificed at two-day intervals. After mice were euthanized, total blood per mouse was collected in a serum separator tube (BD Microtainer; Becton Dickinson, Sparks, MD, USA), and the serum was isolated with centrifugation at 15,000× *g* for 2 min. Cecum samples were immediately placed at –80 °C for extracting DNA, and the distal end of the colon was fixed in 10% buffered formalin phosphate (Sigma-Aldrich, St. Louis, MO, USA) solution for histological observations. Mouse fecal samples were collected on alternate days and maintained in a deep freezer until subsequent assessment.

### 2.2. Fecal Inflammatory Markers

Mouse fecal samples from each group were divided into three groups, completely suspended in DW at a ratio of 50 mg feces/mL by vigorous vortexing, and centrifuged at 10,000× *g* for 3 min to separate the fecal supernatants. The fresh supernatants were aliquoted and kept at −20 °C. Mouse lipocalin-2/NGAL (Lcn2) and myeloperoxidase (MPO) activity in the fecal supernatants were quantified by commercially available ELISA kits (R&D Systems, Minneapolis, MN, USA) in accordance with the manufacturer’s instructions. Optical density was detected at 450 nm and 570 nm, readings at 570 nm were subtracted from the readings at 450 nm, and the final concentration was expressed as ng/g feces.

The amount of hemoglobin (Hgb) from stool was determined according to our previously established luminol method [28]. In brief, the luminol reagent was prepared immediately prior to detection to prevent signal reduction, with the following chemicals (Sigma-Aldrich, St. Louis, MO, USA): 0.1 g of luminol, 5.0 g of Na_2_CO_3_, 15 mL of 30% (*w*/*w*) H_2_O_2_ solution, and 100 mL of DW. Lyophilized human Hgb (Sigma-Aldrich, St. Louis, MO, USA) was used as a standard solution. Luminescence was immediately measured by a SpectraMax M4 Microplate/Cuvette Reader (Molecular Devices, San Jose, CA, USA) after mixing the fecal supernatant and Hgb standard with the same amount of luminol reagent. The whole process was protected from direct light. The final concentration of hemoglobin in feces was expressed as mg/g stool. All detection was conducted in technical duplicate repeats of biological triplicate samples.

### 2.3. Serum Inflammatory Cytokines

Six different inflammatory cytokine (IL-6, IL-10, MCP-1, TNF, IFN-γ, and IL-12p70) levels in mouse serum were determined using a Cytometric Bead Array Mouse Inflammation Kit (Becton Dickinson, Sparks, MD, USA) in accordance with the manufacturer’s protocol. Each specific antibody conjugated to capture beads and its target analyte combined to form sandwich complexes with PE-conjugated detection antibodies. Fluorescence signals were measured using flow cytometry (FACS Canto II; Becton Dickinson, Sparks, MD, USA), and the data were analyzed with Flow Cytometric Analysis Program Array software (version 3.0, Becton Dickinson, Sparks, MD, USA).

### 2.4. Histological Observations

The fixed distal colon end tissues were paraffin embedded and stained with hematoxylin and eosin (H&E) by the Contract Research Organization of LOGONE Bio Convergence Research Foundation (Seoul, South Korea).

### 2.5. DNA Extraction and Purification

Due to the inhibitory effect of DSS on polymerase activity [37,38], we established the optimal conditions to remove DSS from DSS-contaminated cecum samples by dividing the process of obtaining purified DNA into four steps, as follows: (1) the mouse cecum was roughly chopped with dissecting scissors, homogenized, and washed three times with ice-cold purified DW using centrifugation (24,000× *g*) at 4 °C for 7 min; (2) total DNA was extracted according to the QIAamp DNA Mini Kit (Qiagen, Hilden, Germany) protocol; (3) the extracted DNA was precipitated with ice-cold ethanol and 3 mol/L sodium acetate (Sigma-Aldrich, St. Louis, MO, USA), maintaining the entire process on ice [39]; and (4) DNA templates were purified using a QIAquick PCR purification kit (Qiagen, Hilden, Germany) for the removal of residual DSS contaminants. Then, we confirmed that there was no inhibitory effect on DNA polymerase in qRT-PCR when all procedures were performed in sequence. All procedures were equally applied to the cecum samples of the group not treated with DSS.

### 2.6. 16S rRNA Gene Amplification and HiSeq Sequencing

Sequencing libraries of the V3 and V4 regions of the 16S bacterial rRNA gene were constructed following Illumina’s instructions [40]. Briefly, the amplification was performed in a two-step PCR with two PCR clean-up steps. The 16S rRNA amplicons were amplified using V3–V4 region primers: forward [5′-TCGTCGGCAGCGTCAGATGTGTATAAGAGACAGCCTACGGGNGGCWGCAG-3′] and reverse [5′-GTCTCGTGGGCTCGGAGATGTGTATAAGAGACAGGACTACHVGGGTATCTAATCC-3′]. After the amplicon PCR (95 °C 3 min; 25 cycles of 95 °C 30 s, 55 °C 30 s and 72 °C 30 s; and then 72 °C 5 min), the amplicons were cleaned with AMPure XP beads. The second PCR was performed using the purified PCR templates and Nextera XT index primers, and then the PCR products were cleaned before Illumina HiSeq sequencing (Illumina Inc., San Diego, CA, USA) by Macrogen (Seoul, South Korea).

### 2.7. Data Processing, Taxonomic Classification, and Diversity Studies

The 16S rRNA sequence reads were processed in the Quantitative Insight into Microbial Ecology 2 (QIIME 2) pipeline [41]. A total of 39,278,297 demultiplexed sequences with an average of 370,550 reads per sample, ranging from 40,903 to 847,758 reads, were obtained. After the low-quality regions were trimmed and truncated in the DADA2 plugin, samples with a total number of reads below the feature count of 10,000 were removed. Rooted and unrooted phylogenetic trees were created, and taxonomy was assigned using the naive Bayes classifier against the Greengenes reference database (13_8 99% operational taxonomic units (OTUs) from the 515F/806R region of the sequences). Some individual sample sequences were excluded from the beginning of data processing because of low sequence quality. Raw sequences and metadata are available in the public database GenBank (ID PRJNA615701). The assigned OTUs were rarefied to 10,000-read sampling depth, and the OTU table is available in Supplementary Data S1.

To conduct downstream data analysis, the preprocessed files (.qza) from QIIME 2 were imported into R (www.r-project.org) via the qiime2R package, and we generated the phyloseq object to be analyzed in phyloseq R package [42,43]. Alpha diversity was measured with the Chao1 and Shannon indices, and the generalized UniFrac (GUniFrac) method was applied for the calculation of phylogenetic distances [44,45]. The sample distribution was analyzed by a nonmetric multidimensional scaling (NMDS) method, and an ADONIS permutation-based statistical test was conducted in R to determine whether the separation of the sample groups was significant. Data visualization was performed in GraphPad Prism (version 8.3.0; GraphPad software Inc., San Diego, CA, USA).

### 2.8. Colitis-Associated Microbiota Analysis

To identify the particular taxa that increased or decreased in proportion to an increase in dosage and duration of DSS administration, the relative abundances (RAs) of individual groups were agglomerated at the genus level using the *tax_glom* and *merge_sample* method in the phyloseq R package (Supplementary Data S2). The subsequent workflow is summarized as follows: (1) Pearson correlation analysis with exposure duration was applied in each DSS dose group (Supplementary Data S3); (2) the genera showing a strong positive correlation with duration were sorted (Pearson correlation coefficient, PCC > 0.7 and *p* < 0.05) (Supplementary Data S4); (3) the genera showing a strong negative correlation with duration were sorted (PCC < −0.7 and *p* < 0.05) (Supplementary Data S5); and (4) selected genera that have a significant correlation with duration in a serial dose manner were identified as follows: all dose groups, the 2% and 3% DSS groups or only the 3% DSS group (Supplementary Data S6). In order to find the relations of the microbial taxa to colitis severity, fecal Lcn2, MPO, and Hgb levels were used as inflammatory indicators. The correlation between the RAs of the agglomerated OTUs at the genus level and each biomarker was assessed (PCC > 0.6 or < −0.6) with statistical significance (*p* < 0.05).

### 2.9. Statistical Analyses

One-way analysis of variance (ANOVA) was applied to evaluate differences in discrete variables among the groups. The significance of the tested fecal biomarkers was analyzed by two-way ANOVA with two independent variables, and simple linear regression at the 95% confidence interval was performed. Pearson correlation analysis was conducted via two-tailed tests with 95% confidence intervals. Statistical analysis was conducted in GraphPad Prism, except ADONIS in R.

### 2.10. Data Availability

The 16S rRNA gene sequences were deposited in the GenBank Sequence Read Archive (SRA) database (ID PRJNA615701).

## 3. Results

### 3.1. DSS-Driven Colitis Development in a Two-Factor Designed in Vivo Mouse Model

**Figure 1:**
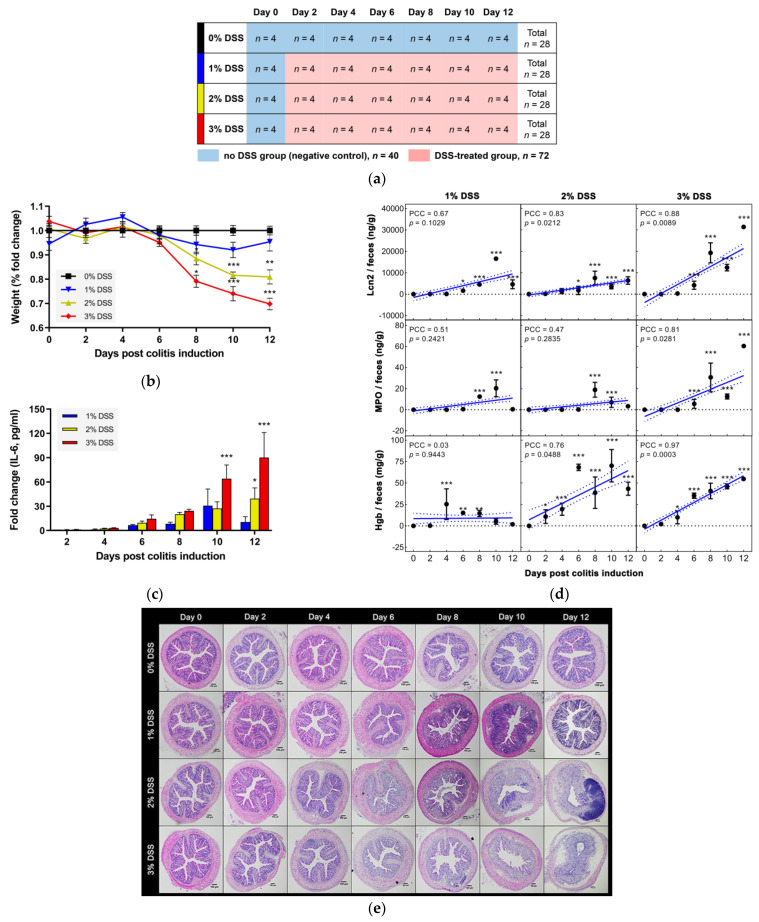
Experimental design and pathological/pathophysiological changes. (**a**) The in vivo experimental scheme. Four mice from each dextran sulfate sodium (DSS) group (total *n* = 28 per group) were sacrificed at two-day intervals. Shown in red is the negative control consisting of the 0% DSS group and the initial mice of all DSS groups. (**b**) Fold change in body weight (%) compared to that of the 0% DSS group. (**c**) Fold change in serum IL-6 level compared to that of the negative control. (**d**) Fecal inflammatory biomarkers. (**e**) Histological architecture of distal colon tissue in DSS-induced colitis mice. Original magnification, 4×. Scale bars, 100 µm. Significance is indicated compared to the 0% DSS group values. Correlations with treatment duration and significance are indicated as PCC and *p* value. All data represent the mean ± SEM. Statistical significance is indicated as follows: * *p* < 0.05, ** *p* < 0.01, and *** *p* < 0.001.

An in vivo experiment was carried out in which four mice were sacrificed at two-day intervals in each DSS dose group (Figure 1a). The experiment was discontinued on day 12 based on the Institutional Animal Care and Use Committee (IACUC) recommendations because losses of 18.0% (*p* < 0.0001) and 31.4% (*p* < 0.0001) of the initial body weight were observed in the 2% and 3% DSS groups, respectively. The negative controls, i.e., the DSS-untreated group, consisted of the 0% DSS group and the initial samples from all DSS-treated groups. Exposure to more than 2% DSS induced a significant loss of body weight from 8 days post colitis induction (Figure 1b). The body weight of mice treated with 1% DSS showed a variation with the day of DSS administration but was not significantly different from that of the negative control group mice. An invasive marker, serum IL-6 tended to increase gradually with treatment duration at concentrations above 2% DSS (Figure 1c). On the other hand, in the 1% DSS group, the IL-6 level was elevated until day 10 and then attenuated on the subsequent test day. Notable results in serum IL-10, MCP-1, TNF, IFN-γ, and IL-12p70 levels were not observed (Supplementary Table S1).

The DSS-induced colitis model, unlike the 2,4,6-trinitrobenzene sulfonic acid (TNBS) model, is suitable for investigating the host immune response because immune cells, such as neutrophils and myeloid cells, are involved [20]. Lcn2 and MPO associated with neutrophil granules were used as noninvasive fecal biomarkers to evaluate the severity of gut inflammation in real time [46]. Compared to that of the negative control, the significant activity of Lcn2 was determined beginning on day 6, while MPO activity showed significant elevation beginning on day 8, except for in the 3% DSS group (Figure 1d). Administration of 3% DSS severely induced both Lcn2 and MPO levels after six days, showing strong positive correlations with treatment duration (PCC = 0.8804 and 0.8075, respectively).

The presence of blood in stool is one of the typical clinical symptoms in colitis [47,48]. We quantified the amount of Hgb in feces based on a luminescence assay. Fecal Hgb in the 1% DSS group showed no correlation with the colitis induction period (PCC = 0.0328), while strong correlations were observed in the 2% and 3% DSS groups (PCC = 0.7570 and 0.9714, respectively) (Figure 1d). Based on our results, Lcn2 and MPO were suitable as biomarkers to differentiate between the 2% and 3% DSS groups, whereas Hgb was sensitive to distinguishing colitis conditions at concentrations below 2% DSS.

DSS-induced colonic mucosal damage was observed longitudinally (Figure 1e). In the negative controls, intact architecture was observed, displaying well-defined gland lengths, no leukocyte infiltration within the mucosa or submucosa, and no ulceration. The 2% and 3% DSS groups showed mild mucosal inflammatory cell infiltrates and focal ulceration with few crypts on day 4. The pathology developed severely from day 6, showing extensive ulceration with dense infiltrate of neutrophils into the mucosa/submucosa and loss of goblet cells. Treatment with 1% DSS resulted in inflammatory cell infiltration into the submucosa with crypt loss on day 6 and increased the infiltrates into the mucosa until day 10. On day 12, in particular, the 1% DSS group had moderately damaged mucosal architecture, including submucosal infiltrates, partial ulceration, and aberrant crypts with mild goblet cell loss. The 1% DSS group result supports the tendency of the fecal and serum biomarkers.

### 3.2. DSS-Driven Microbial Diversity Shifts

**Figure 2:**
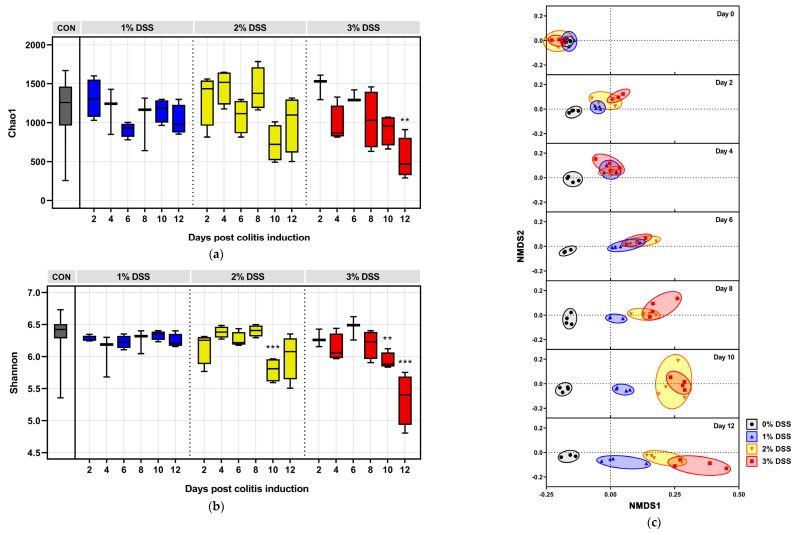

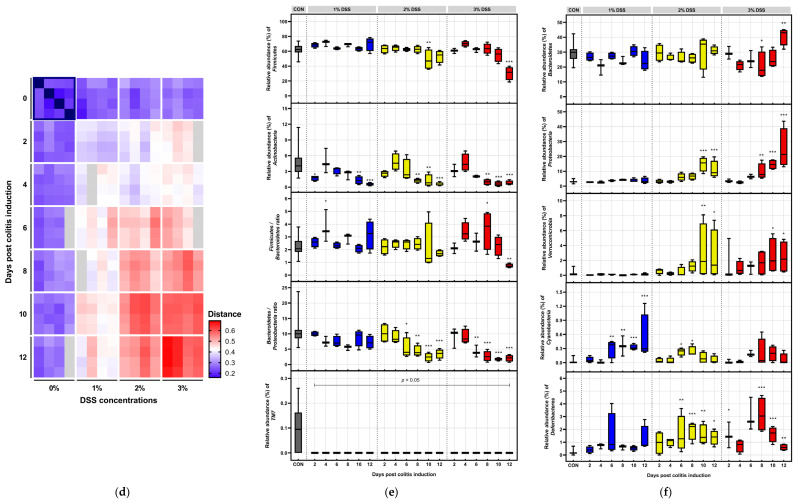
DSS-driven changes in microbial diversity, phylogenetic ordinations, and populations at the phylum level. Alpha diversity estimated as (**a**) Chao1 and (**b**) Shannon indices during DSS colitis induction. (**c**) Nonmetric multidimensional scaling (NMDS) plot of GUniFrac distances. (**d**) Pairwise distance of individuals compared to the initial 0% DSS subjects in a black-lined box. (**e**) Phyla (*Firmicutes* and *Actinobacteria*) and population ratios (*Firmicutes*/*Bacteroidetes* and *Bacteroidetes*/*Proteobacteria*) that decreased with increasing DSS exposure and the phylum whose abundance diminished (*TM7*) with DSS treatment are shown. (**f**) Phyla (*Bacteroidetes*, *Proteobacteria*, *Verrucomicrobia, Cyanobacteria,* and *Deferribacteres*) that increased with increasing DSS exposure. Statistical significance compared to the negative control values is indicated as follows: * *p* < 0.05, ** *p* < 0.01, and *** *p* < 0.001.

To unravel the alterations in microbial abundance and diversity induced by DSS, the Chao1 (F = 2.616 and *p* = 0.0016) and Shannon indices (F = 6.104 and *p* < 0.0001) were analyzed (Figure 2a,b). Treatment with 3% DSS significantly decreased Chao1 scores on day 12 and Shannon index after day 10 compared to those of the negative control (*n* = 38). In the 2% DSS group, the Chao1 and Shannon indices showed a marked tendency to decrease after ten days. On the other hand, neither of the indices were significantly reduced in the 1% DSS group. Significant separation of the individual groups was determined with the ADONIS permutation-based statistical test with 999 permutations (*R*^2^ = 66.4% and *p* = 0.001). In NMDS analysis, dissimilarity between the 0% DSS and DSS-treated groups was observed from day 2, and considerable separation of each dose group was found after eight days of colitis induction (Figure 2c). The cluster of the DSS groups shifted gradually from the 0% DSS group as time passed, and distinct dose-dependent separation was observed on day 12. The pairwise distances between individual samples and initial samples of the 0% DSS group (*n* = 4) showed the dysbiotic condition against normal status (Figure 2d). Detailed data are available in Supplementary Data S7.

### 3.3. Microbial Dynamics Depending on DSS Exposure

At the phylum level, *Firmicutes* and *Actinobacteria* demonstrated a duration-dependent decrease in abundance (Figure 2e), while *Bacteroidetes*, *Proteobacteria*, and *Verrucomicrobia* showed a significant duration-dependent increase in abundance (Figure 2f). Among the other phyla that accounted for low abundance, the RA of *Cyanobacteria* increased in the 1% DSS group (Figure 2f). The populations of *Deferribacteres* in all the DSS dose groups had a similar pattern, rising for 6–8 days and then decreasing subsequently (Figure 2f). The phylum *TM7* was found in only the negative control. The ratio of *Bacteroidetes* and *Proteobacteria* was significantly decreased upon DSS exposure at concentrations above 2% from day 6, and the correlations with duration were analyzed as follows: PCC = −0.9284, *p* = 0.0075 and *R*^2^ = 0.8620 for the 2% DSS group, and PCC = −0.9178, *p* = 0.0099, and *R*^2^ = 0.8424 for the 3% DSS group (Figure 2e). The ratio of *Firmicutes* and *Bacteroidetes* had no considerable correlation with DSS duration, resulting in PCCs of −0.6572 (*p* = 0.1562 and *R*^2^ = 0.4319) and −0.4783 (*p* = 0.3373 and *R*^2^ = 0.2288) in the 2% and 3% DSS groups, respectively (Figure 2e). Notably, the phyla *Firmicutes*, *Bacteroidetes*, *Proteobacteria*, and *Verrucomicrobia* were susceptible to intensive DSS exposure, while *Actinobacteria* showed high susceptibility to even mild DSS exposure.

**Figure 3:**
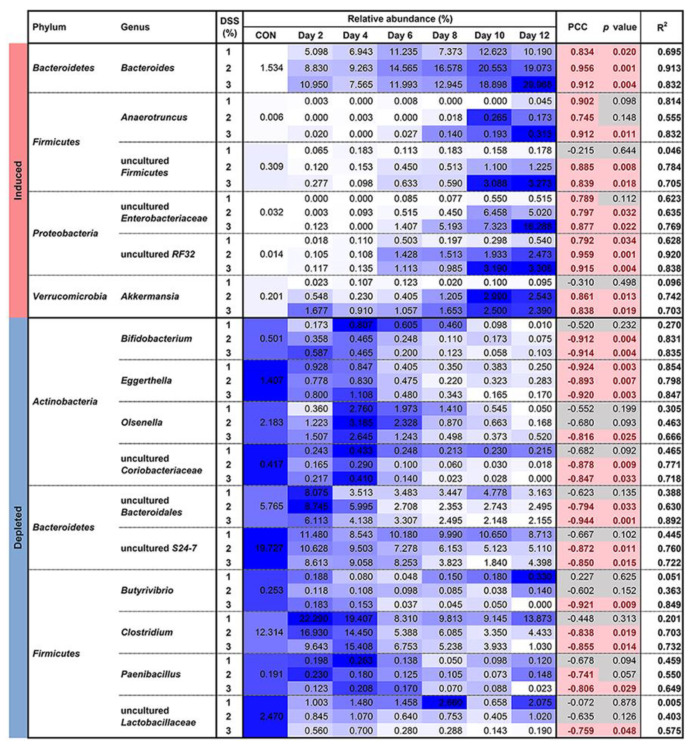
Changing genera depending on DSS exposure. Pearson correlation between the resulting relative abundances (RAs) and DSS duration are indicated. Red represents PCC > 0.7 or <−0.7 with *p* value < 0.05. Blue gradients represent the intensity of RAs within each genus.

The agglomerated RAs were analyzed to identify genera changed in response to DSS dose and duration (Supplementary Data S2) instead of finding the differential abundances using R packages such as DESeq2 [49] and edgeR [50]. The time-series experimental workflow of the DESeq2 vignette was not suitable due to the reductions in log2 fold change estimates and low sample numbers (*n* = 4 per condition) [51,52]. Detailed information about the selection course in this study is available in Supplementary Data S3–S6. As a result, a total of 16 genera were selected depending on DSS exposure (Figure 3). Uncultured order *RF32*, *Bacteroides*, *Akkermansia*, uncultured *Firmicutes, Enterobacteriaceae*, and *Anaerotruncus* were selected as induced genera. Ten genera were selected as depleted genera, as follows: *Eggerthella*, *Bifidobacterium*, uncultured *Coriobacteriaceae*, *Bacteroidales*, *S24-7*, *Clostridium*, *Olsenella*, *Butyrivibrio*, *Paenibacillus*, and uncultured family *Lactobacillaceae*. Genera belonging to the phyla *Proteobacteria* and *Verrucomicrobia* were identified in only the induced genera, while genera belonging to *Actinobacteria* were found in only the depleted genera. The selected uncultured *Enterobacteriaceae*, *RF32*, and *Akkermansia* contributed mainly to the significant enrichment of each phylum (Figure 3 and Figure S1d,e). Notably, the genera *Bifidobacterium*, *Eggerthella*, *Olsenella*, and uncultured family *Coriobacteriaceae* led to a significant reduction in the phylum *Actinobacteria* abundance (Figure 3 and Figure S1c). The genera belonging to the phyla *Bacteroidetes* and *Firmicutes* were classified in both induced and depleted categories. The induction of *Bacteroides* considerably contributed to the enrichment of *Bacteroidetes* despite the opposite contributions of uncultured *Bacteroidales* and *S24-7* (Figure S1b).

### 3.4. Microbial Susceptibility to Colitis Severity

**Figure 4:**
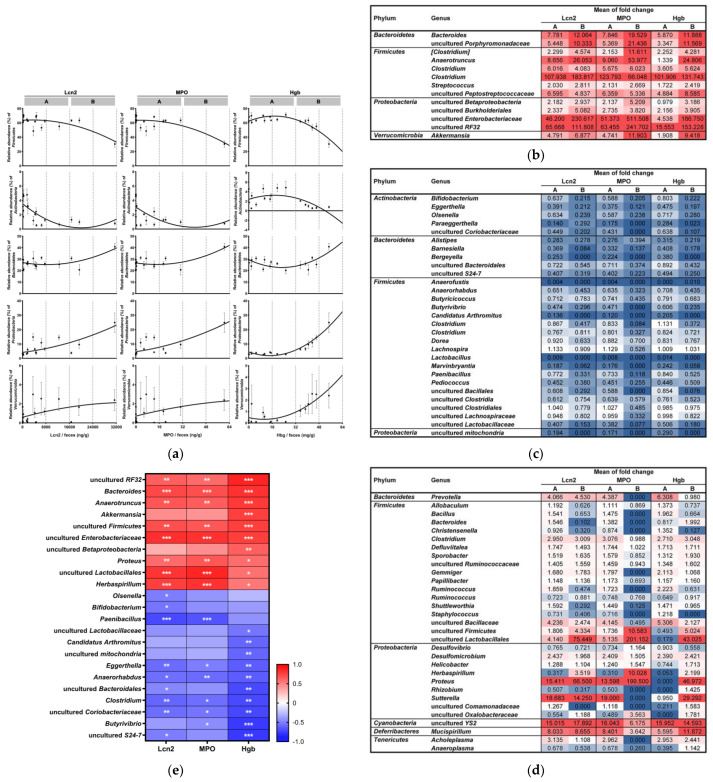
Dynamics of the gut microbiota based on microbial susceptibility to colitis severity. (**a**) Dynamics of microbial changes at the major phylum level. Constantly (**b**) increasing or (**c**) decreasing genus populations from mild to severe status, respectively. (**d**) Taxonomic groups showing dynamic fluctuations from mild to severe status. Color gradients indicate, as follows: compared to the negative control, blue represents decreased populations (fold change < 1.0), red represents increased populations (fold change > 1.0), and white shows no changes (fold change = 1.0). (**e**) Correlation between the resulting RAs and colitis indicators. Statistical significance is indicated as follows: * *p* < 0.05, ** *p* < 0.01, and *** *p* < 0.001.

The cecal microbial communities showed different susceptibility to colitis severity. As colitis intensified, the dynamics of the phylum populations were observed (Figure 4a). The crucial point is that each phylum has a different susceptibility to the colitis status caused by DSS. We divided two sections, ‘A’ and ‘B,’ assigned to the mild and severe colitis status of each index, respectively. There were no considerable changes in the RA of *Firmicutes* in section A of all indices, but *Firmicutes* abundance considerably declined under severe conditions (section B). *Actinobacteria* was more susceptible to mild status, with a drastic decline in section A of Lcn2 and MPO, not Hgb (PCC = −0.4507 and *p* = 0.0694). In contrast, *Verrucomicrobia* was sensitive to severe status of section B, with a strong Hgb association (PCC = 0.7603 and *p* = 0.0004), not Lcn2 and MPO. *Bacteroidetes* showed higher susceptibility to intense inflammation in section B. The RA of *Proteobacteria* consistently increased as Lcn2 and MPO levels increased regardless of the sections, but the enrichment of the phylum was accelerated in section B.

To determine the dynamics of susceptibility to the severity of inflammation at the genus level, the mean of agglomerated RAs of the individual groups over those of the negative control group in each section (Figure 4b–d) was calculated (Supplementary Data S8–S10). Some genera showed continuous increasing or decreasing abundances under mild colitis (section A) and severe colitis (section B) over those in the negative control group (Figure 4b,c). However, certain genera did not show a consistent shift, and some had opposite patterns in the A and B sections (Figure 4d). These results elucidate that the susceptibilities of the bacterial taxonomic groups to colitis status are different and suggest the possibility of obtaining the mismatched results among cross-sectional studies performed with varying colitis status.

The relationship between the microbial composition changes and the colitis conditions remains unclear (Figure 4e). According to the correlation criteria (PCC > 0.6 or <−0.6) with the indicators associated with immune-related proteins (Lcn2 and MPO) and bleeding (Hgb), a total of 20 genera were selected (Supplementary Data S11). The selected genera included the induced or depleted genera depending on DSS exposure (shown in Figure 3), except *Bifidobacterium*, *Olsenella*, and uncultured *Lactobacillaceae*. *Bifidobacterium* and *Olsenella* were not selected based on our PCC criteria, but they had significant correlations with Lcn2 (PCC = −0.5545 and *p* = 0.0137, PCC = −0.5159 and *p* = 0.0238, respectively). Likewise, uncultured *Lactobacillaceae* had a significant correlation with Hgb (PCC = −0.5421 and *p* = 0.0246). *Bacteroides*, *Anaerotruncus*, uncultured *Firmicutes*, and uncultured *Enterobacteriaceae* showed significant relationships with all indices. Of note, *Candidatus Arthromitus*, *Akkermansia*, uncultured *Betaproteobacteria*, and uncultured *mitochondria* were significantly related to only Hgb, whereas *Paenibacillus* was correlated only to the neutrophil-derived indicators (Lcn2 and MPO).

Overall, this study demonstrated that colitis development and concomitant decreases in intestinal microbial diversity depend on the degree of DSS exposure, but changes in the gut microbial composition are based on the susceptibility of each taxon to colitis severity. Furthermore, the correlations between taxa and colitis indicators provide an understanding of gut dysbiosis in IBD pathogenesis.

## 4. Discussion

Gut homeostasis refers to a microbial composition that is stable and balanced with the host immune system [3]. Disturbances in this homeostasis lead to a pathogenic state called dysbiosis by depleting beneficial microbes and changing microbial diversity [53,54]. In general, decreased populations of *Faecalibacterium*, *Clostridiales*, *Lactobacillus*, and *Bifidobacterium* and increased populations of *Enterobacteriaceae* and *Escherichia coli* within the class *Gammaproteobacteria* have been reported in IBD patients, but there are inconsistencies among clinical cases [16,17,18].

In DSS-induced colitis mice, *Enterobacteriaceae* and *Bacteroides* are well known to expand in the inflamed intestine, including in our results (Figure 3 and Figure 4e). However, there is obvious heterogeneity in microbiome results among studies (Table S2), which can result from mouse genetic and environmental factors, as well as research methods [6,35,55,56]. In particular, a cross-sectional study design is one of the main causes [35]. For instance, in this study, *Paenibacillus* and *Anaerotruncus* might not be considered DSS colitis-associated taxa if the study focused on a single time point on day 6 with 2% DSS (Figure 3). In addition, *Olsenella* may also be excluded from the DSS colitis taxa if we compared its RA at day 6 with that in the negative control (Figure 3).

In previous longitudinal metagenomic investigations in IBD patients and colitis animals, the dynamics of the intestinal microbiota have been observed, which were linked to the course of disease development [12,19,57,58]. In the present study, by dissecting the intestinal microbiota based on mild and severe status, it was shown that individual taxonomic groups have specific susceptibility to colitis severity (Figure 4a–d). As representative results, the population of *Herbaspirillum* showed opposite tendencies in mild (section A) and severe (section B) conditions (Figure 4d). Moreover, some taxa (e.g., *Proteus* and uncultured *Lactobacillales*) had opposite patterns between the sections depending on Hgb (Figure 4d).

Colitis-associated microbe identification remains elusive. We tried to analyze the restructured bacterial taxa depending on DSS exposure and confirmed their correlations with colitis severity (Figure 3 and Figure 4e). As reported in previous studies (Supplementary Table S2), *Bacteroides*, *Akkermansia*, and *Enterobacteriaceae* showed consistent expansions in the inflamed gut (Figure 3 and Figure 4e). Current assumptions to explain this result are as follows. (i) High oxygen levels confer superiority in the consumption of nutrients and niche occupation to *Enterobacteriaceae* over strict anaerobes (such as *Clostridium* and *Bifidobacterium*), and *Enterobacteriaceae* growth is enhanced by the utilization of sialic acids degraded by *Bacteroides* from mucin as a nutrient [32,59,60]. (ii) Lcn2, a secreted inflammatory cytokine from epithelial and myeloid cells, binds to the iron-chelating siderophore enterobactin (Ent) released by gut bacteria such as *E. coli* and *Salmonella*. It inhibits Ent-mediated iron acquisition and supports the bactericidal enzyme MPO [61]. However, *Enterobacteriaceae* pathogens do not rely solely on Ent for iron uptake, as they produce another form of siderophores [62]. (iii) Hgb from intestinal bleeding may play a key role in increasing the *Akkermansia* population in the mucosal layer, leading to colonic mucolysis [63]. The increased Hgb levels also induce gut dysbiosis by increasing gram-negative bacteria and directly exacerbate colitis through free radical production, cytotoxicity induction, and lipid peroxidation reaction catalysis [30]. We confirmed the strong correlation between *Akkermansia* and fecal Hgb (Figure 4e). The damaged mucosal architectures in the 2% and 3% DSS groups were regarded as supplying Hgb to some mucus-degrading bacteria (Figure 1d,e). Recent studies have suggested that administration of *Akkermansia muciniphila* attenuated inflammation in DSS-induced colitis mice and that these potential probiotic properties would allow *A. muciniphila* use as a therapeutic agent [29]. In our opinion, however, the application of *A. muciniphila* in diseases accompanied by gut bleeding should be carefully considered based on a sufficient number of studies.

A novel focus in our investigation is the bacterial taxa, which have a significant correlation with Hgb or neutrophil-derived biomarkers (Figure 4e). *Candidatus Arthromitus* was annotated from the Greengenes database, but the original lineage was *Candidatus Savagella* within the *Clostridiaceae* family, known as segmented filamentous bacteria (SFB) [64]. As commensals in the mammalian gut, SFB penetrate the intestinal mucus layer without invading epithelial cells. They do not trigger host immunity that induces secretory IgA production and stimulates T cell responses (Th1, Th17, and Treg cells) [65,66,67]. Interestingly, *Candidatus Arthromitus* disappeared with the Hgb-enhanced expansion of mucus-degrading bacteria, such as *Akkermansia* and *Bacteroides* (Figure 4e). Furthermore, *Paenibacillus* was the only genus that had strong correlations with Lcn2 and MPO but not Hgb (Figure 4e). The genus *Paenibacillus* is a gram-variable, facultative anaerobic, and endospore-forming bacteria with low G + C genomic features. The genus was originally included within the genus *Bacillus* but reclassified to *Paenibacillus* based on phylogenetic analysis with 16S rRNA sequences in 1993 [68]. Notably, it has been well characterized that *Paenibacillus* produces a wide range of antimicrobial compounds against pathogens [69] and bacillibactin, which is structurally similar to Ent, as a possible Lcn2 ligand [70]. This could support the negative relationship of *Paenibacillus* populations with Lcn2′s companion MPO and Lcn2 (Figure 4e).

The relation of the toxic effect of hydrogen sulfide produced by sulfate-reducing bacteria (SRB), mainly *Desulfovibrio* spp., with ulcerative colitis has been reported [71,72]. The amount of sulfate and pH in the intestine influence the abundance of SRB leading to intestinal inflammation in the distal colon [73,74]. However, there are heterogeneous results for the SRB population in the cross-sectional DSS colitis model [75,76,77], and our longitudinal analysis showing the dynamics of *Desulfovibrio* and *Desulfomicrobium* support the inconclusive results (Figure 4d). According to Laroui et al. [23], individual DSS-associated molecules (free glucose, sulfate, and free dextran) did not induce significant colitis in mice independently, and intact DSS chemicals linked to medium-chain-length fatty acids activated the inflammatory pathway in colonic epithelial cells.

Whether altering the gut microbiota is a cause or consequence of colitis is an ongoing debate. According to Eichele et al. [27], some studies have reported that reduced gut microbial diversity occurred early prior to the physiological observations of inflammation. On the other hand, our results demonstrated that the microbial diversity gradually shifted from that of the negative control group and intensified under severe DSS exposure (Figure 2a–d), showing almost parallel pathological/pathophysiological evidence (Figure 1b–e). Moreover, we found that microbial composition changes showed various dynamics depending on the individual taxonomic group (Figure 2e,f, Figure 3 and Figure 4a–d). Consequently, it can be postulated that disease development in the DSS colitis murine model results from mutual and simultaneous interactions among the mucus barrier, intestinal microflora, and host immune response.

The metagenome sequencing technology has expanded our knowledge and information on microbial compositions in ecology. Its function has also been enabled to analogize by other meta-omics technologies, such as metabolomics, metatranscriptomics, and metaproteomics. However, the computational methods do not overcome the strain-level resolution [78,79]. In addition, there are still a lot of unknown taxa of the gut microbiome [80,81]. Although we selected the colitis-associated genera by a two-factor design rather than a single time point or simple long-term analysis, there are limitations in revealing specific strain resolution and unknown taxa, described as ‘uncultured’ (Figure 3 and Figure 4e). As a future direction, to unravel the functions of gut microbes in IBD pathogenesis, culturable gut microorganisms should be expanded and accumulated more than currently obtained [81,82]. Eventually, a deep observation and understanding of the gut microbiota at strain-level resolution could reveal host–microbe and microbe–microbe interactions in specific intestinal conditions.

## 5. Conclusions

In summary, we demonstrated colitis-associated genera by considering intestinal microbial dynamics based on serial DSS dose and duration, and confirmed their susceptibilities to the gut inflamed conditions. Our findings revealed that microbiota composition in colitis intestine is dynamic due to individual taxonomic groups having their own susceptibility to changing colitis status, and specific populations showed significant correlations with intestinal bleeding and/or neutrophil-derived indicators. To elicit the underlying mechanisms of microbial susceptibility in IBD pathogenesis, more comprehensive and specific information on the gut microbes should be provided from culture-based approaches (high-throughput culturomics and target-specific reverse genomics) with integrated omics technologies [83,84].

## Data Availability

The 16S rRNA gene sequences were deposited in the GenBank Sequence Read Archive (SRA) database (ID PRJNA615701).

## Supplementary Materials

The following are available online at https://www.mdpi.com/2076-2607/9/2/370/s1, Data S1: Relative abundance OTU table of cecal samples (*n* = 106) with taxonomic assignments, Data S2: Relative abundance of the agglomerated genera in each group (merged samples), Data S3: Pearson correlation between relative abundance and colitis induction days, Data S4: The genera showing a positive correlation with DSS treatment duration, Data S5: The genera showing a negative correlation with DSS treatment duration, Data S6: Selection of genera that changed in a dose- and duration-dependent manner, Data S7: GUniFrac distance of individual samples, Data S8: Calculation of the mean of agglomerated RAs of the individual groups over those of the negative control in Lcn2 sections, Data S9: Calculation of the mean of agglomerated RAs of the individual groups over those of the negative control in MPO sections, Data S10: Calculation of the mean of agglomerated RAs of the individual groups over those of the negative control in Hgb sections, Data S11: Pearson correlation between resulting RAs and colitis biomarkers, Table S1: Serum cytokine (pg/mL) changes after colitis induction, Table S2: Comparison of changed gut microbial taxa in DSS-induced colitis mice compared with those in healthy controls, Figure S1: Agglomerated RAs (%) of bacterial genera for each phylum. (a) *Firmicutes*, (b) *Bacteroidetes*, (c) *Actinobacteria*, (d) *Proteobacteria*, (e) *Verrucomicrobia*, (f) *TM7*, (g) *Cyanobacteria*, (h) *Deferribacteres*, and (i) *Tenericutes*.
