# Supplementary material for: Longitudinal Microbiome Analysis in a Dextran Sulfate Sodium-Induced Colitis Mouse Model
Source: Microorganisms. 2021 Feb 12;9(2):370. doi: 10.3390/microorganisms9020370 (PMC7917662; doi:10.3390/microorganisms9020370)
Supplement: Supplementary file 1 [file microorganisms-09-00370-s001.zip › Park et al., Supplementary data/Park et al., Supplementary Figure and Table.pdf]

microorganisms

MDPI

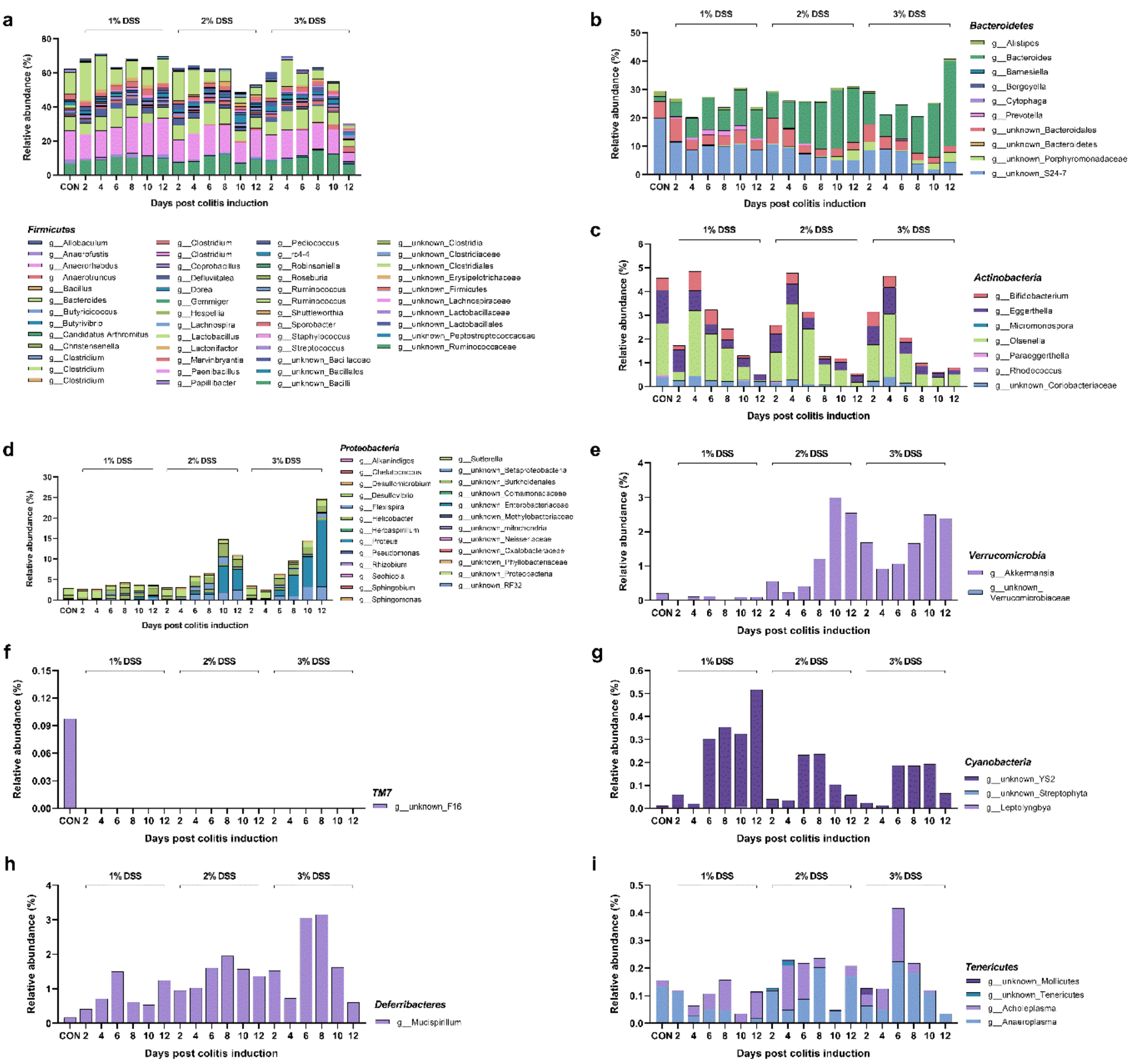

Figure S1. Agglomerated RAs (%) of bacterial genera for each phylum. (**a**) *Firmicutes*, (**b**) *Bacteroidetes*, (**c**) *Actinobacteria*, (**d**) *Proteobacteria*, (**e**) *Verrucomicrobia*, (**f**) *TM7*, (**g**) *Cyanobacteria*, (**h**) *Deferribacteres*, and (**i**) *Tenericutes*.

Table S1. Serum cytokine (pg/ml) changes after colitis induction

Cytokine	DSS (%)	Fold change					
		Day 2	Day 4	Day 6	Day 8	Day 10	Day 12
**IL-6**	1	1.04 ± 0.00	1.27 ± 0.66	6.50 ± 0.98	8.28 ± 1.95	30.75 ± 20.55	10.48 ± 6.56
	2	0.84 ± 0.29	2.33 ± 0.53	9.40 ± 2.18	20.16 ± 2.36	27.25 ± 8.25	39.34 ± 13.31*
	3	1.26 ± 0.14	3.07 ± 0.30	14.53 ± 4.80	24.31 ± 1.90	63.96 ± 16.89***	90.18 ± 30.89***
**MCP-1**	1	0.90 ± 0.26	1.12 ± 0.10	1.19 ± 0.15	1.15 ± 0.12	2.26 ± 0.58***	1.67 ± 0.36
	2	1.05 ± 0.17	0.89 ± 0.11	1.20 ± 0.40	1.86 ± 0.37*	1.64 ± 0.50	1.43 ± 0.29
	3	1.07 ± 0.14	1.37 ± 0.22	1.14 ± 0.22	2.06 ± 0.28**	1.66 ± 0.29	0.74 ± 0.42
**TNF**	1	1.56 ± 0.48	0.93 ± 0.33	1.43 ± 0.51	1.70 ± 0.38	2.26 ± 0.70	1.45 ± 0.48
	2	1.06 ± 0.24	1.55 ± 0.35	2.10 ± 0.39	2.52 ± 0.64*	1.55 ± 0.43	2.62 ± 0.63*
	3	1.28 ± 0.41	1.32 ± 0.39	1.40 ± 0.25	2.04 ± 0.11	2.21 ± 0.72	1.97 ± 0.64

Data is expressed as the mean ± standard deviation. Fold change on negative control. Significance is indicated as follows: * $p < 0.05$, ** $p < 0.01$ and *** $p < 0.001$. Serum IL-10, IFN-γ, and IL-12p70 were all below detection limits in this assay.

Table S2. Comparison of changed gut microbial taxa in DSS-induced colitis mice compared with those in healthy controls.

Sample	DSS colitis induction	Analysis method	*Firmicutes*	*Bacteroidetes*	*Actinobacteria*	*Proteobacteria*	*Verrucomicrobia*	*Deferribacteres*	Reference
Cecum	C57BL/6J female<br>1.5% DSS for 8 days<br>followed by 8 days of recovery	16s rRNA amplicon sequencing<br>Illumina Miseq<br>Greengenes database	- *Firmicutes*<br>↓ *Allobaculum*<br>↓ *Butyricoccus*<br>↓ *Dorea*<br>↓ *Lactobacillus*<br>↑ *Clostridium*<br>↑ *rc4-4*<br>↑ *Ruminococcus*<br>↑ uncultured *Bacillaceae*<br>↑ uncultured *Lachnospiraceae*	- *Bacteroidetes*<br>↑ *Bacteroides*	↓ *Actinobacteria*<br>↓ *Bifidobacterium*<br>↓ *Eggerthella*<br>↓ *Olsenella*	- *Proteobacteria*<br>↑ uncultured *Enterobacteriaceae*	↑ *Verrucomicrobia*	N.A.	[1]
Cecum	C57BL/6<br>5% DSS for 14 days	16s rRNA amplicon sequencing<br>Ribosomal Database<br>Project (RDP) classifier	↑ *Firmicutes*<br>↓ *Ruminococcaceae*<br>↑ *Lachnospiraceae*	↓ *Bacteroidetes*	N.A.	↓ *Proteobacteria*	↑ *Verrucomicrobia*	N.A.	[2]
Stool	C57BL/6J male<br>2% DSS for 7 days<br>followed by 2 days of recovery	16s rRNA amplicon sequencing<br>Ion S5™ XL platform<br>Silva database	↓ *Firmicutes*<br>↓ *Bacilli*<br>↓ *Lactobacillales*<br>↓ *Lactobacillaceae*<br>↓ *Lactobacillus*<br>↓ *Lachnospiraceae*<br>↓ *Alistipes*<br>↑ *Erysipelotrichales*<br>↑ *Erysipelotrichaceae*<br>↑ *Erysipelotrichia*<br>↑ *Turicibacter*	↑ *Bacteroidetes*<br>↓ *Rikenellaceae*<br>↑ *Porphyromonadaceae*<br>↑ *Bacteroidaceae*<br>↑ *Bacteroides*<br>↑ *Odoribacter*<br>↑ *Prevotellaceae*	N.A.	↑ *Proteobacteria*<br>↑ *Gammaproteobacteria*<br>↑ *Enterobacteriales*<br>↑ *Enterobacteriaceae*<br>↑ *Escherichia/Shigella*	N.A.	N.A.	[3]
Stool	C57BL/6 female<br>0.75% DSS for 10 days	16s rRNA amplicon sequencing<br>Illumina Miseq<br>Greengenes database	↓ *Firmicutes*<br>↓ uncultured *Mogibacteriaceae*<br>↓ *rc4-4*<br>↓ uncultured *Christensenellaceae*<br>↓ uncultured *Clostridiaceae*<br>↓ uncultured *Clostridiales*<br>↓ *SMB53* sp.<br>↓ *Clostridium perfringens*<br>↓ *Blautia* sp.<br>↓ *Lactobacillus* sp.	↓ *Bacteroidetes*<br>↓ uncultured *Rikenellaceae*<br>↓ uncultured *S24-7*	N.A.	↑ *Proteobacteria*<br>↑ *uncultured Enterobacteriaceae*<br>↑ *Escherichia coli*	N.A.	N.A.	[4]
Stool	C57BL/6J male<br>3%-3.5% DSS for 5 days	16s rRNA pyrosequencing<br>Roche 454 GS-FLX system<br>Ribosomal Database<br>Project (RDP) classifier & Greengenes database	↓ *Lachnospiraceae*<br>↓ *Ruminococcaceae*<br>↓ *Alistipes*<br>↓ *Anaeroplasma*<br>↓ *Oscillibacter*<br>↑ *Erysipelotrichaceae*<br>↑ *Lactobacillaceae*<br>↑ *Lactobacillus*	↓ *Porphyromonadaceae*<br>↓ *Prevotellaceae*<br>↓ *Rikenellaceae*<br>↓ *Barnesiella*<br>↓ *Parabacteroides*<br>↓ *Prevotella*<br>↑ *Bacteroidaceae*<br>↑ *Bacteroides*	N.A.	↓ *Parasutterella*<br>↑ *Enterobacteriaceae*<br>↑ *Helicobacteraceae*<br>↑ *Escherichia/Shigella*	N.A.	N.A.	[5]
Stool	C57BL/6J male and female<br>3% DSS for 7 days<br>followed by 2 days of recovery	16s rRNA amplicon sequencing<br>Illumina Miseq<br>Ribosomal Database<br>Project (RDP) classifier	↓ *Firmicutes*<br>↓ *Alistipes*<br>↓ *Lactobacillus*<br>↓ *Intestinimonas*<br>↓ *Pseudoflavonifactor*<br>↓ *Oscillibacter*<br>↓ *Acetatifactor*<br>↓ *Dorea*<br>↓ *Enterohabdus*<br>↓ *Clostridium XIVa*<br>↓ *Clostridium IV*<br>↓ *Clostridium XIVb*<br>↓ *Flavonifactor*	↑ *Bacteroidetes*<br>↓ *Alloprevotella*<br>↓ *Odoribacter*<br>↓ *Prevotella*<br>↓ *Barnesiella*<br>↑ *Parabacteroides*<br>↑ *Bacteroides*	N.A.	↓ *Proteobacteria*<br>↓ *Helicobacter*<br>↑ *Escherichia/Shigella*	↑ *Verrucomicrobia*<br>↑ *Akkermansia*	N.A.	[6]

Table S2. Continued

Sample	DSS colitis induction	Analysis method	*Firmicutes*	*Bacteroidetes*	*Actinobacteria*	*Proteobacteria*	*Verrucomicrobia*	*Deferribacteres*	Reference
			↓ *Sporobacter*<br>↓ *Anaeroplasma*<br>↑ *Clostridium XVIII*<br>↑ *Clostridium XI*<br>↑ *Turicibacter*<br>↑ *Romboutsia*<br>↑ *Clostridium sensu stricto*						
Luminal contents	C57BL/6N<br>2% DSS for 7 days<br>followed by 2 days of recovery	16s rRNA pyrosequencing<br>Roche 454 GS-FLX system<br>Ribosomal Database Project (RDP) classifier	↓ *Alistipes*<br>↑ *Enterococcus*<br>↑ *Lactobacillus*<br>↑ *Lactobacillales*<br>↑ *Ruminococcaceae*<br>↑ *Clostridales*<br>↓ ↑ *Lachnospiraceae*	↓ *Bacteroidales*<br>↑ *Bacteroides*<br>↑ *Parabacteroides*<br>↑ *Prevotella*<br>↓ ↑ *Porphyromonadaceae*	N.A.	↑ *Enterobacteriaceae*	↑ *Akkermansia*	↑ *Mucispirillum*	[7]
Stool	CBA/J female<br>4% DSS for 16 days	16s rRNA amplicon sequencing<br>Illumina Miseq<br>Silva database	↓ uncultured *Clostridales*<br>↓ *Alistipes*<br>↓ uncultured *Lachnospiraceae*<br>↓ *Anaerotruncus*<br>↑ *Staphylococcus*<br>↑ *Acetitomaculum*<br>↑ *Coprococcus*<br>↑ uncultured *Ruminococcaceae*<br>↑ *Ruminococcaceae IncertaeSedis*<br>↑ *Lachnospiraceae IncertaeSedis*	↑ uncultured *S24-7*	N.A.	N.A.	↑ *Akkermansia*	N.A.	[8]
Stool	C57BL/6J female<br>3% DSS for 7 days<br>followed by 7 days of recovery	16s rRNA amplicon sequencing<br>Illumina Miseq	↓ *Allobaculum*<br>↓ *Clostridium*	↓ *Porphyromonas*	↓ *Actinobacteria*<br>↓ *Bifidobacterium*<br>↓ *Olsenella*	↑ *Proteobacteria*<br>↑ *Escherichia*	↓ *Verrucomicrobia*<br>↓ *Akkermansia*	N.A.	[9]
Colon	C57BL/6J male<br>3% DSS for 7 days<br>followed by 1 days of recovery	16s rRNA amplicon sequencing<br>Illumina Hiseq<br>Ribosomal Database Project (RDP) classifier	↓ *Clostridia*<br>↓ *Clostridiales*<br>↓ *Lachnospiraceae*<br>↑ *Enterococcus*<br>↑ *Enterococcaceae*<br>↑ *Enterococcus faecalis*<br>↑ *Peptostreptococcaceae*<br>↑ *Clostridium sensu stricto*<br>↑ *Clostridiaceae*	↓ *Bacteroidetes*<br>↓ *Bacteroidia*<br>↓ *Bacteroidales*<br>↓ *Prevotellaceae*<br>↓ *Bacteroidales S24-7* group<br>↓ *Prevotella*		↑ *Proteobacteria*<br>↑ *Enterobacteriaceae*<br>↑ *Gammaproteobacteria*<br>↑ *Enterobacteriales*<br>↑ *Escherichia/Shigella*<br>↑ *Escherichia coli*		↑ *Deferribacteres*<br>↑ unculutred *Deferribacteres*<br>↑ *Deferribacterales*<br>↑ *Deferribacteraceae*<br>↑ *Mucispirillum*<br>↑ *Mucispirillum schaedleri*	[10]
Stool	CBA/J female<br>5% DSS for 8-9 days	Culture-dependent method	↓ *Eubacterium* sp.<br>↓ *Enterococcus* sp.<br>↑ *Clostridium* sp.	↑ *Bacteroidaceae*	N.A.	↑ *Enterobacteriaceae*	N.A.	N.A.	[11]
Colon	C57BL/6 female<br>4% DSS for 7 days	qRT-PCR with specific primers	↓ *Lactobacillus*	N.A.	N.A.	↑ *Enterobacteriaceae*<br>↑ *Desulfovibrio*	↑ *Akkermansia*	N.A.	[12]

Arrows indates the increased or decreased populations compared to the abundances in no DSS treatement group. -: no difference; N.A.: not applicable.

References

1. Yeo, S.; Park, H.; Seo, E.; Kim, J.; Kim, B.K.; Choi, I.S.; Huh, C.S. Anti-inflammatory and gut microbiota modulatory effect of *Lactobacillus rhamnosus* strain LDTM 7511 in a dextran sulfate sodium-induced colitis murine model. *Microorganisms* **2020**, *8*, 845.
2. Nagalingam, N.A.; Kao, J.Y.; Young, V.B. Microbial ecology of the murine gut associated with the development of dextran sodium sulfate-induced colitis. *Inflamm. Bowel Dis.* **2011**, *17*, 917–26.
3. Bian, X.; Wu, W.; Yang, L.; Lv, L.; Wang, Q.; Li, Y.; Ye, J.; Fang, D.; Wu, J.; Jiang, X.; et al. Administration of *Akkermansia muciniphila* ameliorates dextran sulfate sodium-induced ulcerative colitis in mice. *Front. Microbiol.* **2019**, *10*, 2259.
4. Constante, M.; Fragoso, G.; Calvé, A.; Samba-Mondonga, M.; Santos, M.M. Dietary heme induces gut dysbiosis, aggravates colitis, and potentiates the development of adenomas in mice. *Front. Microbiol.* **2017**, *8*, 1809.
5. Huang, Y.L.; Chassard, C.; Hausmann, M.; von Itzstein, M.; Hennet, T. Sialic acid catabolism drives intestinal inflammation and microbial dysbiosis in mice. *Nat. Commun.* **2015**, *6*, 8141.
6. de Bruyn, M.; Sabino, J.; Vandeputte, D.; Vermeire, S.; Raes, J. Comparisons of gut microbiota profiles in wild-type and gelatinase B/matrix metalloproteinase-9-deficient mice in acute DSS-induced colitis. *npj Biofilms Microbi.* **2018**, *4*, 18.
7. Berry, D.; Schwab, C.; Milinovich, G.; Reichert, J.; Ben Mahfoudh, K.; Decker, T.; Engel, M.; Hai, B.; Hainzl, E.; Heider, S.; et al. Phylotype-level 16S rRNA analysis reveals new bacterial indicators of health state in acute murine colitis. *ISME J.* **2012**, *6*, 2091–2106.
8. Borton, M.A.; Sabag-Daigle, A.; Wu, J.; Solden, L.M.; O'Banion, B.S.; Daly, R.A.; Wolfe, R.A.; Gonzalez, J.F.; Wysocki, V.H.; Ahmer, B.M.M.; et al. Chemical and pathogen-induced inflammation disrupt the murine intestinal microbiome. *Microbiome* **2017**, *5*, 47.
9. Lee, K.W.; Kim, M.; Lee, C.H. Treatment of dextran sulfate sodium-induced colitis with mucosa-associated lymphoid tissue lymphoma translocation 1 inhibitor MI-2 Is associated with restoration of gut immune function and the microbiota. *Infect. Immun.* **2018**, *86*, e00091-18.
10. Li, M.; Wang, B.; Sun, X.; Tang, Y.; Wei, X.; Ge, B.; Tang, Y.; Deng, Y.; He, C.; Yuan, J.; et al. Upregulation of intestinal barrier function in mice with DSS-induced colitis by a defined bacterial consortium is associated with expansion of IL-17A producing gamma delta T cells. *Front. Immunol.* **2017**, *8*, 824.
11. Okayasu, I.; Hatakeyama, S.; Yamada, M.; Ohkusa, T.; Inagaki, Y.; Nakaya, R. A novel method in the induction of reliable experimental acute and chronic ulcerative colitis in mice. *Gastroenterology* **1990**, *98*, 694–702.
12. Håkansson, Å.; Tormo-Badia, N.; Baridi, A.; Xu, J.; Molin, G.; Hagslätt, M.L.; Karlsson, C.; Jeppsson, B.; Cilio, C.M.; Ahrné, S. Immunological alteration and changes of gut microbiota after dextran sulfate sodium (DSS) administration in mice. Clin. Exp. Med. **2015**, *15*, 107–120.
